# Metal Oxide Wrapped by Reduced Graphene Oxide Nanocomposites as Anode Materials for Lithium-Ion Batteries

**DOI:** 10.3390/nano13020296

**Published:** 2023-01-11

**Authors:** Junaid Aslam, Yong Wang

**Affiliations:** 1 Department of Chemical Engineering, School of Environmental and Chemical Engineering, Shanghai University, 99 Shangda Road, Shanghai 200444, China; 2 Key Laboratory of Organic Compound Pollution Control Engineering (MOE), Shanghai University, 99 Shangda Road, Shanghai 200444, China

**Keywords:** Fe_2_O_3_, Co_3_O_4_, reduced graphene oxide, ball milling, lithium-ion batteries, anode material

## Abstract

The reduced graphene oxide/iron oxide (rGO/Fe_2_O_3_) and reduced graphene oxide/cobalt oxide (rGO/Co_3_O_4_**)** composite anodes have been successfully prepared through a simple and scalable ball-milling synthesis. The substantial interaction of Fe_2_O_3_ and Co_3_O_4_ with the rGO matrix strengthens the electronic conductivity and limits the volume variation during cycling in the rGO/Fe_2_O_3_ and rGO/Co_3_O_4_ composites because reduced graphene oxide (rGO) helps the metal oxides (MOs) to attain a more efficient diffusion of Li-ions and leads to high specific capacities. As anode materials for LIBs, the rGO/Fe_2_O_3_ and rGO/Co_3_O_4_ composites demonstrate overall superb electrochemical properties, especially rGO/Fe_2_O_3_T−5 and rGO/Co_3_O_4_T−5, showcasing higher reversible capacities of 1021 and 773 mAhg^−1^ after 100 cycles at 100 mAg^−1^, accompanied by the significant rate performance. Because of their superior electrochemical efficiency, high capacity and low cost, the rGO/Fe_2_O_3_ and rGO/Co_3_O_4_ composites made by ball milling could be outstanding anode materials for LIBs. Due to the excellent electrochemical performance, the rGO/Fe_2_O_3_ and rGO/Co_3_O_4_ composites prepared via ball milling could be promising anode materials with a high capacity and low cost for LIBs. The findings may provide shed some light on how other metal oxides wrapped by rGO can be prepared for future applications.

## 1. Introduction

Nowadays, LIBs are one of the primary trends for next-generation electric automobiles and energy storage equipment due to their high energy density, extended cycle performance and good specific capacity [1,2]. However, due to its low theoretical capacity (372 mAhg^−1^), the typical graphite-based anode cannot meet the continuously growing need for better energy storage batteries [3]. Therefore, we have a significant need to create alternative anode materials with improved electrochemical properties [4,5]. Many efforts have been made to study the nature and structure of the electrodes of LIBs [6]. Several potential materials have been explored as potential electrode candidates for LIBs, such as silicon [7]. The silicon-based electrodes maintain a high specific capacity but suffer from the aging process at large cycles due to the volume expansion and pulverization of Si [8,9]. The morphology changes with cycling, the structural changes followed by the aging process and the mechanical robustness of Si nanoparticles for applications in lithium-ion batteries have also been widely investigated in order to obtain more knowledge about this potential material [10,11,12]. Many people are interested in transition metal oxides (TMOs) because of their high theoretical capacities, ample natural reserves and affordable prices [13]. In this field, various types of materials have been explored, e.g., Co_3_O_4_ [14], Fe_2_O_3_ [15], SnO_2_ [16], MnO_2_ [17], Sb_2_Se_3_ [18], CuO [19] and Ni_3_Se_2_ [20]. Because of their rich redox reactions, which involve numerous ions and generate high specific capacities, TMOs have been regarded as one of the most viable electrode materials for LIBs [21,22]. Fe_2_O_3_ and Co_3_O_4_ are two TMOs with large theoretical capacities that are anticipated to meet the needs of future energy storage systems (approximately 1000 and 890 mAhg^−1^), respectively. Nevertheless, like many other TMOs, these compounds still have a number of drawbacks, including poor intrinsic electrical conductivity, ineffective ion transport kinetics and conspicuous volume expansion/contraction during the charge/discharge processes [23,24,25]. 

By combining carbonaceous materials with Fe_2_O_3_ and Co_3_O_4_ to improve electrical conductivity and decrease the volume expansion during a charge/discharge, as well as to improve cycling stability, these problems can be effectively addressed. Several studies also reported that the problems related to the structural degradation of potential anode materials, such as Si, were partially mitigated by introducing carbonaceous materials; for example, Si/graphite composites were proved to be the significant anode materials for LIBs [26,27,28]. Carbon-based materials, such as graphene [29], carbon nanotube [30] and porous carbon [31], have been extensively researched in recent years by being doped with the anode materials of LIBs. Because of its high specific surface area and highly conductive skeleton, rGO has attracted the most attention among these carbon materials and has been used as a conductive additive in a variety of energy storage devices, including supercapacitors [32], sodium-ion batteries [33] and lithium-sulfur batteries [34]. As a result, rGO is thought to be more advantageous for decorating various metals and metal oxide nanoparticles on graphene sheets [35]. Recently, a number of research studies on Fe_2_O_3_ and Co_3_O_4_ composites containing rGO have been published. For example, Xu et al. fabricated flexible anodes with a good rate performance by using porous Fe_2_O_3_/graphene nano-frameworks [36]. Yang et al. fabricated hollow Fe_2_O_3_ nanoparticles with N-doped graphene aerogels, showcasing a high reversible capacity (1483 mAhg^−1^ at 0.1 Ag^−1^) [37]. Subsequently, due to its high specific capacity (830.7 mAhg^−1^ after 75 cycles at 200 mAg^−1^) and outstanding electrochemical reactivity, Lou, Y. et al. investigated Co_3_O_4_ in LIBs as a highly suitable anode material [38].

In this paper, we used ball milling to establish the rGO/Fe_2_O_3_ and rGO/Co_3_O_4_ composites with good cyclic performance and rate capability. The force provided by the high-speed collision of stainless steel balls during ball milling to break graphitic layers (crack C−C bonds and covalent bonds) assisted the Fe_2_O_3_ and Co_3_O_4_ particles in forming a strained and stable structure with the rGO [39]. Moreover, we have synthesized rGO/Fe_2_O_3_ and rGO/Co_3_O_4_ with five different ratios, as shown in Table 1. The rGO/Fe_2_O_3_T−5 and rGO/Co_3_O_4_T−5 nanocomposites showcase higher reversible capacities of 1021 and 773 mAhg^−1^ after 100 cycles at a current density of 100 mAg^−1^ and demonstrate a significant rate capacity at various current densities. On the one hand, highly conductive rGO provides pathways for fast electron transportation, increasing the reversible capacities of Fe_2_O_3_ and Co_3_O_4_; on the other hand, highly conductive rGO prevents the volume change in Fe_2_O_3_ and Co_3_O_4_ during repeated discharge–charge cycles. As a result, the capacity and cycling performance of rGO/Fe_2_O_3_ and rGO/Co_3_O_4_ are significantly enhanced.

## 2. Materials and Methods

### 2.1. Chemicals

The chemicals used were as follows: Graphite powder, Co_3_O_4_ powder (30 nm, 99%, Aldrich, Darmstadt, Germany), Fe_2_O_3_ (30 nm, 99%, Aldrich), sulfuric acid (95%), potassium permanganate (KMnO_4_), hydrochloric acid, hydrogen peroxide, Hydrazine, toluene, polyvinylidene fluoride (PVdF), N-methylpyrrolidone (NMP) (Sigma Aldrich, Darmstadt, Germany), copper foil (10 mm thickness, Schlenk Metallfolien, Germany), electrolyte 1M lithium hexafluorophosphate solution (LiPF_6_) in ethylene carbonate (EC): diethyl carbonate (DEC): ethyl methyl carbonate (EMC) (1:1:1, by weight, Danvec, Singapore) and Celgard 2320 separator.

### 2.2. Synthesis of GO/rGO

The manufacturing of rGO involves three major steps: (1) oxidation, (2) exfoliation of the manufactured products and (3) the reduction of GO into rGO. In the first step, graphite oxide was prepared from graphite powder using a modified Hummers process. In a standard technique, 4g of graphite powder was added to 69 mL of concentrated H_2_SO_4_, and 9g of KMnO_4_ was slowly and continuously stirred into the aforesaid mixture. Because a fast temperature rise might create an explosion, the ice bath was utilized as a precaution to cool the reaction mixture, and the temperature was kept below 20 °C. Then, 150 mL of distilled water was added, causing a rapid temperature increase to 100 °C. An amount of 500 mL of distilled water and 30 mL of H_2_O_2_ were poured after 1 h, and the color turned yellow. The resultant suspension was washed with 800 mL of diluted HCl (1:10). Metal ions were removed using filtration, and the finalized product was placed in a 60 °C oven to dry under vacuum. Water was used to dissolve the resultant clump, which was then washed many times until the pH reached 7. The washed product was dispersed in water through ultrasonication and centrifuged at 4000 rpm for 30 min, resulting in the removal of exfoliated GO. The exfoliated GO was dried for 2 h at 60 °C. The GO was then converted to rGO using N_2_H_4_ in the following phase. In this phase, GO is dispersed in distilled water (0.5 mg/mL) and then sonicated for 1 h at temperatures below 50 °C. Then, in a Teflon-lined autoclave, 2 mL of N_2_H_4_ was added to 60 mL of GO. In order to make rGO, the autoclave was heated for 12 h at 180 °C, after which the material was cleaned and dried.

### 2.3. Synthesis of Fe_2_O_3_/Co_3_O_4_-Doped rGO 

**Figure 1:**
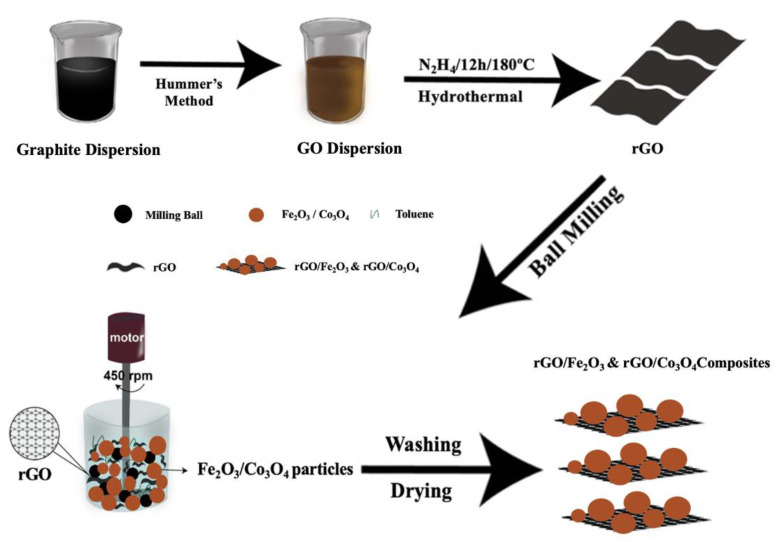
Schematic representation of the rGO/Fe_2_O_3_ and rGO/Co_3_O_4_ nanocomposites’ synthesis process.

To produce the rGO/Fe_2_O_3_ and rGO/Co_3_O_4_ nanocomposites, reduced graphene oxide: iron oxide and reduced graphene oxide: cobalt oxide were sealed in a stainless vial in varying weight ratios (90:10, 80:20, 70:30, 60:40 and 50:50) alternatively and ball milled for 20 h at 450 rpm. Once it had settled down, the solvent was separated by centrifugation, and then the mixture was ground. The resulting mixture was vacuum dried for 24 h at 60 °C. In order to obtain the samples for characterization, the as-prepared materials were centrifuged three times at 1000 rpm to collect the resultant product. The resultant product was then cleaned with distilled water and dried in the oven at 60 °C for 24 h. The distilled water was clear after washing, which indicated that most of the raw black rGO remained in the final obtained sample. In addition, it is worth mentioning that there is a large scope in controlling the size distribution because the milling conditions during the ball milling process greatly influence the particle size and size distribution of the resulting product. The size distribution of the particles can be controlled by controlling the milling conditions, such as mill speed, ball filling, slurry filling and slurry concentration. Therefore, the particle size and size distribution can be controlled by changing the milling conditions during ball milling [40]. Figure 1 displays a schematic representation of the synthesis technique. 

### 2.4. Material Characterization 

X-ray diffraction was performed using XRD (R-Hitachi, Tokyo, Japan). The materials’ form, size and texture were examined using transmission electron microscopy (TEM; J-200C, Tokyo, Japan) and scanning electron microscopy (SEM; J-6700, Tokyo, Japan). Oxford-X energy dispersive X-ray spectroscopy was used to examine the element distribution in the nanocomposites (EDX). Raman spectroscopy was performed using a Renishaw-X Plus source, and the thermogravimetric analysis (TGA; STA 409 P-NETZSCH) was performed in an air environment with an optimal heating rate (10 °C/min). The chemical structures and bonding of the synthesized samples were confirmed using Fourier-transform infrared spectroscopy (FTIR; IS50 FT-IR), and the electrochemical impedance studies (EIS; CHI660D, 0.01Hz–100 kHz, 5 mVs^−1^) were utilized to characterize the manufactured anode materials. Brunauer–Emmett–Teller (BET; ASAP 2020M^+^CMICROMERITICS) was used to compute the specific surface area, and the pore size distribution was calculated using the Barrett–Joyner–Halenda (BJH) method, which is based on N_2_ adsorption–desorption isotherms.

### 2.5. Electrochemical Evaluation

Two-electrode CR2032-type coin cells were used for the electrochemical tests. The synthesized samples were combined in an optimum weight proportion (8:1:1), where 80 percent of the active material was utilized, with 10% carbon black as a conductive agent and 10% PVDF as a binder, and then dispersed in deionized water. The resultant slurry was homogeneously placed on a copper foil (13 mm disk) and dried in an oven at an optimum temperature (80 °C) under vacuum for 24 h to serve as the working anode. The active substance has a mass deposition of 1.0–1.2 mg cm^−2^. The coin-type cells were fabricated using electrodes: a lithium foil reference electrode and a Celgard 2400 polypropylene microporous film in an Ar-filled glove box. Furthermore, 1.0 M LiPF_6_ was thus used as the electrolyte. On an electrochemical workstation (CHI660D) with an optimum scan rate (0.2 mVs^−1^) and a particular potential range, all CV measurements were carried out (0.01–3.0 V). Afterwards, the galvanostatic charging was performed in an optimal voltage range (0.01–3.0 V) with the Li^+^/Li at various current densities (50, 100, 200, 500) mAg^−1^ using a battery test system (LAND-CT2001). The EIS tests were performed under a specific AC amplitude (10 mV) followed by a frequency ranging from 0.01 Hz to 100 kHz.

## 3. Results

### 3.1. Characterization of Anode Materials

#### 3.1.1. Crystallinity and Phase Analysis 

**Figure 2:**
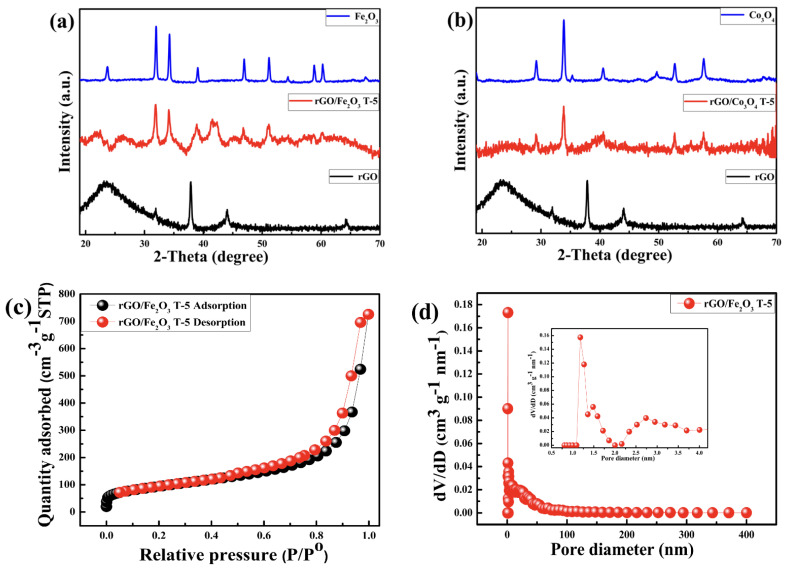
(**a**) X-ray diffraction patterns of the Fe_2_O_3,_ rGO/Fe_2_O_3_T−5 nanocomposite, and the pristine rGO (from top to bottom). (**b**) X-ray diffraction patterns of the Co_3_O_4,_ rGO/Co_3_O_4_T−5 nanocomposite, and the pristine rGO (from top to bottom). (**c**) Nitrogen adsorption-desorption isotherms of the rGO/Fe_2_O_3_T−5 nanocomposite and (**d**) the corresponding pore size distribution of the rGO/Fe_2_O_3_T−5 nanocomposite.

XRD was used to determine the crystallinity and phase purity of the synthesized products for the rGO/Fe_2_O_3_ and rGO/Co_3_O_4_ composites, as shown in Figure 2a,b, alternatively. Figure 2a shows the XRD patterns of rGO, pure Fe_2_O_3_ nanoparticles and the rGO/Fe_2_O_3_T−5 composite. The diffraction peaks of the Fe_2_O_3_ nanoparticles are easily identified. All the strong diffraction peaks are consistent with (012), (104), (110), (113), (024), (018), (214) and (300) of the hematite crystalline Fe_2_O_3_ phase (JCPDS card no. 33-0664). No diffraction peaks of the rGO sheets were seen, revealing that the rGO sheets are fully exfoliated and that the diffraction peaks of rGO are covered by those of Fe_2_O_3_ [41]. The XRD spectra further confirm the successful synthesis of the rGO/Fe_2_O_3_T−5 composite. Moreover, the XRD patterns of the Co_3_O_4_, rGO and rGO/Co_3_O_4_T−5 composites are shown in Figure 2b. The five reflection peaks of Co_3_O_4_ appeared at 2θ = 19.07°, 31.38°, 36.98°, 44.97°, 59.58° and 65.45° and can be assigned, respectively, to the 111, 220, 311, 400, 511 and 440 crystalline planes of cubic Co_3_O_4_ (JCPDS Card 03-065-3103). The rGO only shows a very broad reflection peak at 2θ = 25.40°, which corresponds to a d-spacing of 0.350 nm with an index of 002. This characteristic peak is defined as the rGO sheets that may re-tighten to form a short-range order graphite structure, according to this characteristic peak [42]. Consequently, the XRD patterns show that the ball-milling process was effective in acquiring the Co_3_O_4_ and rGO/Co_3_O_4_T−5 composites.

#### 3.1.2. BET

To obtain the porous nature and Brunauer–Emmett–Teller (BET) specific surface area of the as-prepared samples, the N_2_ adsorption and desorption isotherms were evaluated. As shown in Figure 2c and Figure S1, the adsorption curves of the rGO/Fe_2_O_3_T−5 and rGO/Co_3_O_4_T−5 nanocomposites did not exhibit any adsorption limit at the relatively higher relative pressure (P/P^0^) region. As a result, the sample was classified as a Type IV isothermal model with an H_3_ hysteresis loop by the International Union of Pure and Applied Chemistry [43]. This classification demonstrated that the samples were layered and that the materials were supposed to contain mesopores. This result was confirmed in Figure 2d and Figure S1a, where the pore size distribution for the rGO/Fe_2_O_3_T−5 and rGO/Co_3_O_4_T−5 nanocomposites showed a unimodal-type pore size distribution with peak canters located at 1.23 and 1.24, alternatively. Figure 2d and Figure S1b also showed that the Fe_2_O_3_/rGO materials had not only small-size mesopores but also large-size mesopores. Furthermore, the BET-specific surface areas of the rGO/Fe_2_O_3_T−5 and rGO/Co_3_O_4_T−5 nanocomposites were 372.3917 m^2^/g and 334.351 m^2^/g, respectively.

#### 3.1.3. Raman Spectra

**Figure 3:**
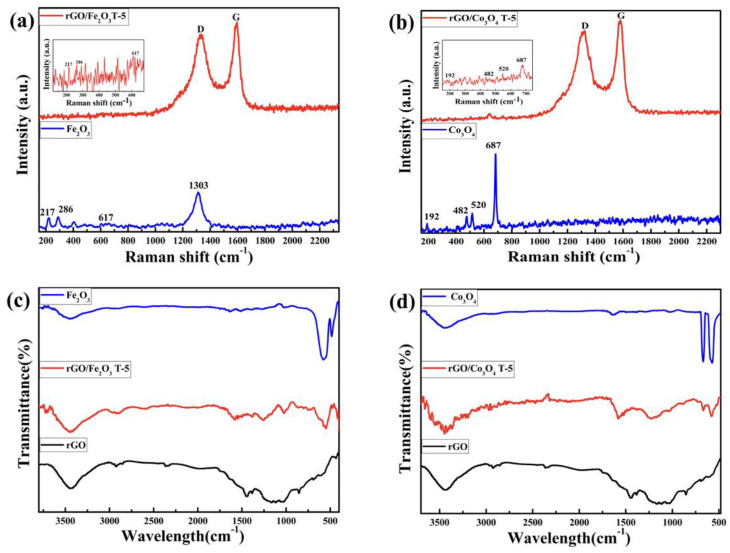
(**a**) Raman spectra of the Fe_2_O_3_ and rGO/Fe_2_O_3_T−5 nanocomposites (from top to bottom). (**b**) Raman spectra of the Co_3_O_4_ and rGO/Co_3_O_4_T−5 nanocomposites (from top to bottom). (**c**) FTIR spectra of the Fe_2_O_3_, rGO/Fe_2_O_3_T−5 nanocomposite and the pristine rGO (from top to bottom). (**d**) FTIR spectra of the Co_3_O_4,_ rGO/Co_3_O_4_T−5 nanocomposite and the pristine rGO (from top to bottom).

The Raman spectra of the rGO/Fe_2_O_3_T−5 nanocomposite and pristine Fe_2_O_3_ are shown in Figure 3a. The Raman spectra of the Fe_2_O_3_ feature two peaks at 217 and 286 cm^−1^, which are most closely attributable to the A_1g_ and E_g_ Raman modes of hematite. Furthermore, a stronger peak appeared around 1303 cm^−1^ due to a two-magnon dispersion of Fe_2_O_3_ [44]. An even weaker peak at 617 cm^−1^ is apparently due to the E_g_ mode of hematite. Furthermore, the rGO/Fe_2_O_3_ nanocomposite exhibits two distinct peaks at 1327 and 1584 cm^−1^, which correspond to the graphene peaks [45,46]. Furthermore, Figure 3a depicts the standard peaks of bare Fe_2_O_3_ as well as the D and G peaks of rGO [47], indicating the amalgamation of Fe_2_O_3_ with rGO. The Raman spectra revealed no impurity peaks, exemplifying the high purity of all the products. The Raman spectra also affirm that the rGO/Fe_2_O_3_ nanocomposite was prepared successfully. Meanwhile, the rGO/Co_3_O_4_T−5 nanocomposite also shows another two obvious peaks at 1327 and 1584 cm^−1^ in Figure 3b, which can be indexed to the peaks of the graphene [45,46]. For Co_3_O_4_, the Raman peaks at 192 cm^−1^ and 520 cm^−1^ were assigned to the F_2g_ mode vibration, and the peaks at 482 cm^−1^ and 687 cm^−1^ were designated to the E_g_ and A_1g_ mode vibrations, respectively [48]. These Co_3_O_4_ vibration peaks can be seen in the rGO/Co_3_O_4_T−5 nanocomposite, which further exemplifies the presence of both rGO and Co_3_O_4_ in the newly synthesized nanocomposite.

#### 3.1.4. FTIR

The FTIR spectra of Fe_2_O_3_, rGO and the rGO/Fe_2_O_3_T−5 nanocomposite are shown in Figure 3c. The absorption peaks at 3746 cm^−1^ and 1384 cm^−1^ for the Fe_2_O_3_ nanoparticles are thought to be due to the symmetric stretching vibration and the bending (in-plane) of the absorbed water’s O–H bond, respectively [49]. As shown in the FTIR spectrum of rGO, the absorption peak of the O–H bonds shifted to 3436.77 cm^−1^, and the decreases in the intensity of the peaks at 1726.84 cm^−1^ (C=O stretching vibrations), 1219.28 cm^−1^ (aromatic C=C) and 1049.42 cm^−1^ (C–O stretching vibrations) show the conversion from GO to rGO [50,51]. In the FTIR spectrum of the rGO/Fe_2_O_3_ nanocomposite, the peaks in the range of 560 cm^−1^ to 470 cm^−1^ could be assigned to the stretching vibrations of Fe–O, which would ensure the successful formation of the rGO/Fe_2_O_3_T−5 nanocomposite. 

The FTIR spectra of rGO, Co_3_O_4_ and the rGO/Co_3_O_4_T−5 nanocomposite are shown in Figure 3d. As shown in the FTIR spectrum of rGO, the absorption peak of the O–H bonds shifted to 3436.77 cm^−1^, and the decreases in the intensity of the peaks at 1726.84 cm^−1^ (C=O stretching vibrations), 1219.28 cm^−1^ (aromatic C=C) and 1049.42 cm^−1^ (C–O stretching vibrations) suggest a transformation from GO to rGO [34,35]. The bands at 661 cm^−1^ and 565 cm^−1^ show the presence of Co–O bonds in the rGO/Co_3_O_4_T−5 nanocomposite [52,53]. The characteristic split vibrational bands of the Co–O bonds and observed O–H stretching at 3449 cm^−1^ in the FTIR spectrum of the Co_3_O_4_ are also consistent in the spectrum of the rGO/Co_3_O_4_T−5 nanocomposite. Except for the peaks due to the Co–O bonds (in Co_3_O_4_), the FTIR spectra of the rGO/Co_3_O_4_T−5 nanocomposite affirmed that the Co_3_O_4_ was satisfactorily packed onto nanosheets of reduced graphene oxide (rGO), with the exception of the peaks due to the Co−O bonds (in Co_3_O_4_) [54].

#### 3.1.5. TGA 

The TGA curves of the rGO/Fe_2_O_3_T−5 and rGO/Co_3_O_4_T−5 nanocomposites are shown in Figure S2. The rGO/Fe_2_O_3_T−5 composite was heated to 800 °C in air. The partial weight loss of the rGO/Fe_2_O_3_T−5 and rGO/Co_3_O_4_T−5 nanocomposites is <8% in the pre-300 °C temperature range, which is most likely due to the adsorbed water evaporation and the extraction of oxygen-containing functional groups from the surface of the rGO sheets. Furthermore, a considerable weight loss occurred owing to rGO’s disintegration and combustion at temperatures ranging from 450 to 550 °C [55]. The results indicate that the percentages of Fe_2_O_3_ and Co_3_O_4_ in the composites rGO/Fe_2_O_3_T−5 and rGO/Co_3_O_4_T−5 are approximately 51.26 wt. % and 44.70 wt. %.

#### 3.1.6. SEM/EDS/TEM

**Figure 4:**
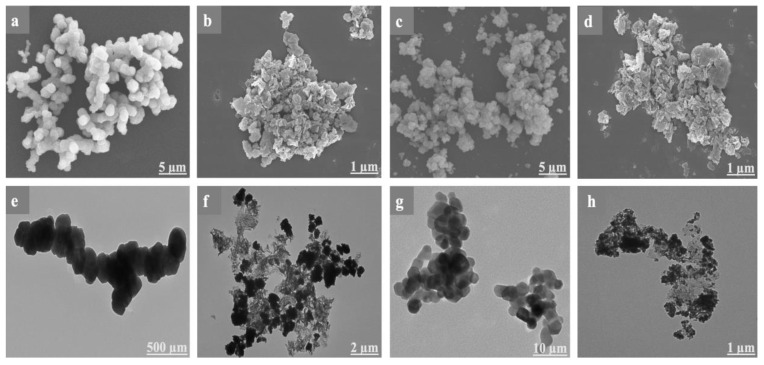
SEM images of (**a**,**b**) Fe_2_O_3_ and the rGO/Fe_2_O_3_T−5, (**c**,**d**) Co_3_O_4_ and rGO/Co_3_O_4_T−5. TEM images of (**e**,**f**) Fe_2_O_3_ and the rGO/Fe_2_O_3_T−5, (**g**,**h**) Co_3_O_4_ and rGO/Co_3_O_4_T−5.

The morphology of the rGO/Fe_2_O_3_T−5 and rGO/Co_3_O_4_T−5 nanocomposites was further clarified using SEM. Figure 4a,c depicts the morphology of the Fe_2_O_3_/Co_3_O_4_ particles. In Figure 4b,d, the SEM images of the rGO/Fe_2_O_3_T−5 and rGO/Co_3_O_4_T−5 nanocomposites indicate the homogenous distribution of Fe_2_O_3_ and Co_3_O_4_ nanoparticles on the surface of the rGO sheets. The deposition of the Fe_2_O_3_ and Co_3_O_4_ nanoparticles on the rGO sheets inhibits them from restacking and maximizes the interlayer space. It can be seen that the Fe_2_O_3_ and Co_3_O_4_ particles (the bright zones) are evenly distributed on the rGO sheets. The Fe_2_O_3_ particles were wrapped by rGO, and a large proportion of the observed Fe_2_O_3_ particles were approximately 27 nm in size. Meanwhile, the Co_3_O_4_ particles wrapped by rGO were less than 25 nm in size. There is a reduction in the particle size of the Fe_2_O_3_ and Co_3_O_4_ nanoparticles, which were approximately 50 nm and 40 nm before ball milling, respectively. The rGO is subdivided into spherical particles by the force of milling balls, and a huge proportion of the Fe_2_O_3_ and Co_3_O_4_ particles were wrapped by them. The interaction of Fe_2_O_3_, Co_3_O_4_ and rGO is further explained using TEM, as shown in Figure 4e–h. Figure 4e,g clearly shows the Fe_2_O_3_ and Co_3_O_4_ particles in the form of dark phases. The bright phases in Figure 4f,h represent rGO, and the dark ones are Fe_2_O_3_ and Co_3_O_4,_ which indicates that the Fe_2_O_3_ and Co_3_O_4_ particles are wrapped by the rGO. After 100 cycles, the rGO/Fe_2_O_3_T−5 and rGO/Co_3_O_4_T−5 electrodes were finally dismantled for the SEM observation. After 100 cycles, the coin cells were dismantled, depicting the presence of the narrow size distribution of the Fe_2_O_3_ and Co_3_O_4_ particles with the help of SEM, as shown in Figure S3, illustrating that graphene can optimize the volume expansion while maintaining the structural strength of a composite. The discharged cells were subsequently dismantled in a glove box after cycling under an argon atmosphere (O_2_, H_2_O < 0.1 ppm) by slicing the two ends of the metal shell with a proper cutting tool to prevent short circuits or any other destructive side reactions during the cell opening. The case was carefully removed. The individual parts of the cell (the outer separator, anode, inner separator and anode) were separated. The extracted samples were not rinsed in order to avoid any type of change/alteration in the morphology of the electrodes. Afterwards, the samples were dried in the glove box. The dried electrode samples were then transferred to the SEM for imaging with the help of a vacuum transfer holder to avoid air exposure. Moreover, Figure S4a–d presents the uniform distribution of the Fe, C and O elements in the rGO/Fe_2_O_3_T−5 composites. Figure S4e–g presents the uniform distribution of the Co, C, and O elements in the rGO/Co_3_O_4_T−5 nanocomposite. Figure S5 shows the SEM image of rGO (a), the TEM image of rGO (b) and the corresponding elemental mapping of (c) C and (d) O.

### 3.2. Electrochemical Performances as Anode Material for LIBs

As working electrodes in LIBs, several ratios of the rGO/Fe_2_O_3_ and rGO/Co_3_O_4_ composites, as well as pristine rGO, were used. The electrochemical characteristics of the synthesized anode materials in relation to the charge–discharge profiles, cycle stability and EIS were investigated to determine their efficacy and efficiency in LIB devices. For this work, 80 percent of the active material, 10% carbon black as a conductive agent, and 10% PVDF as a binder were utilized. In an agate mortar and pestle, fine dry active ingredients were mashed into a slurry. All the components were placed in the mortar pestle and mixed for 30 min before adding NMP dropwise and grinding further until a fine black slurry was formed. Copper was utilized as the current collector, owing to its low potential and excellent endurance. A 3*6-inch copper foil was chopped down and laid flat on a glass bar. The slurry was then coated on the foil. After drying for 6 h in a vacuum oven with the active material, the disks were cut down to construct the LIB coin cells (CR2032) with a Celgard 2400 separator and 1 M LiPF_6_ in EC: DEC: EMC electrolyte. The coin cells were constructed in an argon gas-filled glove box. The electrochemical performance of the active materials was assessed using cyclic voltammetry, impedance measurements and a constant-current discharge charge. At room temperature, the voltage range was tuned to 0.0–3.0 V vs. Li^+^/Li.

#### 3.2.1. Cyclic Voltammetry

**Figure 5:**
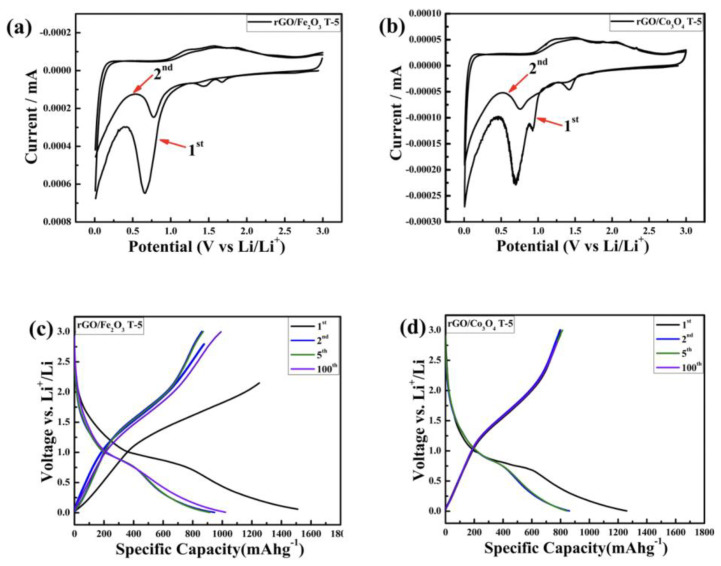
(**a**,**b**) CV results of the rGO/Fe_2_O_3_T−5 and the rGO/Co_3_O_4_T−5 nanocomposites. Galvanostatic charge–discharge profiles: (**c**,**d**) the rGO/Fe_2_O_3_T−5 and the rGO/Co_3_O_4_T−5 electrodes at 1st, 2nd, 5th and 100th cycles at the current density of 100 mAg^−1^.

The electrochemical characteristics of the rGO/Fe_2_O_3_T−5 as the LIB anode were examined using cyclic voltammetry (CV), as shown in Figure 5a. The CV of the rGO/Fe_2_O_3_T−5 was conducted for the first two cycles within an optimal voltage range of 0.0–3.0 V versus Li^+^/Li. In the first cycle, three small peaks between 1.16–1.92 V were observed in the first anodic process of the rGO/Fe_2_O_3_T−5 anode, which can be attributed to the reduction of Fe^0^ to Fe^2+^ and the further oxidation to Fe^3+^ [56]. In the first cycle, there are two peaks at 0.01 and 0.75 V in the cathodic polarization process, which should be ascribed to the creation of a lot of LiC_6_ and the reduction of Fe_2_O_3_ [57]. Meanwhile, the reduction peaks between 1.40–1.70 V in the first cycle of the rGO/Fe_2_O_3_T−5 anode demonstrate lithium intercalation into the Fe_2_O_3_ lattice, forming a transitional phase containing Lix (Fe_2_O_3_). Other researchers have previously reported that when the potential drops below 1.0 V, a solid electrolyte interphase (SEI) and polymeric gel-like layer forms on the electrode’s surface [58,59]. Furthermore, the reversible lithium insertion and extraction processes for the Fe_2_O_3_ nanoparticles and the rGO/Fe_2_O_3_T−5 nanocomposite material may be classified as follows [25,47,48,49]:

Fe_2_O_3_ + xLi^+^ + xe^−^→ Lix (Fe_2_O_3_) (1)

Lix(Fe_2_O_3_) + (6 − x)Li^+^ + (6 − x)e^−^ ↔ 2Fe + 3Li_2_O (2)

In the second cycle, the peak at around 0.70 V shifts to a higher potential (0.80 V), revealing that the reduction of Fe_2_O_3_ occurs much easier after the first cycle. The nanometric metal particles, established during the first discharging process, are kept in the subsequent cycles, resulting in enhanced activity. This phenomenon is frequent in metal oxides [60]. The CV curves of the rGO/Fe_2_O_3_ composite are still almost totally overlapping after the first cycle, indicating that the rGO/Fe_2_O_3_ composite has amazing reversibility.

The electrochemical properties of the rGO/Co_3_O_4_T−5 nanocomposite as LIB anodes were investigated using cyclic voltammetry (CV), as shown in Figure 5b. The CV of the rGO/Co_3_O_4_T−5 nanocomposite was conducted between the first two cycles within an optimal voltage range of 0.0–3.0V versus Li^+^/Li. As shown in Figure 5b, for the rGO/Co_3_O_4_T−5 anode, cathodic peaks between 0.001–1.75 V and anodic peaks between 1.10–2.2 V were clearly observed in the CVs, which are assigned to the development of the clusters between metallic Co and Li_2_O and the reformation of the Co_3_O_4_ phase, respectively, during the conversion reaction [22,61]. As shown in the CV curves, there are multiple cathodic peaks between 0.001–1.75 V witnessed in the first discharge process, corresponding to the multi-step electrochemical reduction between the Li-ions and Co_3_O_4_. Two anodic peaks appeared between 1.10–2.2 V, owing to the oxidation of Co atoms and in accordance with the reported publications [62,63]. The reactions can be described as follows:

Co_3_O_4_ + 8Li^+^ + 8e^−^ → 3Co + 4Li_2_O (3)

Co + Li_2_O → 2Li^+^ + CoO + 2e^−^ (4)

In the second cycle, the cathodic peaks shifted to ~0.75 V, indicating a potential toward stabilization. The sharp cathodic peak claims fast kinetics for the phase transformation of Co_3_O_4_ and the development of the solid electrolyte interphase (SEI) on the surface of the rGO/Co_3_O_4_T−5 nanocomposite, showcasing superior electrochemical reversibility of the rGO/Co_3_O_4_T−5 electrode [64]. 

#### 3.2.2. Galvanostatic Charge–Discharge

The typical properties of the rGO/Fe_2_O_3_ (T−1,T−2,T−3,T−4,T−5) and rGO/Co_3_O_4_ (T−1,T−2,T−3,T−4,T−5) composites were investigated using galvanostatic discharge–charge measurements for the first, second, fifth and hundredth cycles with a current density of 100 mAg^−1^ in the voltage range of 0.01–3.0 V vs. Li^+^/Li. The charge–discharge voltage profiles of the rGO/Fe_2_O_3_T−5 electrode for the first, second, fifth and hundredth cycles with a current density of 100 mAg^−1^ are shown in Figure 5c. The CV results are consistent with the potential plateaus reported in the discharge curves. During the insertion process, the first discharge capacity was 1510 mAhg^−1,^ and the succeeding charge capacity was 1250 mAhg^−1^. The discharge capacity was reduced to 948 mAhg^−1^ during the second cycle, with a comparable charge capacity of 875 mAhg^−1^, resulting in a greater Coulombic efficiency of 92.2%. The discharge capacity was reduced to 915 mAhg^−1^ during the fifth cycle, with a corresponding charge capacity of 864 mAhg^−1^, resulting in a substantially greater Coulombic efficiency of 94.4%. In the 100th cycle, the discharge and charge capacities of the rGO/Fe_2_O_3_T−5 nanocomposite increased to 1021.9 and 990 mAhg^−1^, respectively, indicating a probable activation phase in the electrode material. The lower capacities recorded during the initial few cycles might be attributable to the trapping of Li-ions inside the Fe_2_O_3_ particles’ framework, which are gradually set to release during the cycling, assisting in capacity rise [65]. As a consequence, the Coulombic efficiency rose to approximately 97.09%.

Figure S7 depicts the charge–discharge voltage profiles of the rGO/Fe_2_O_3_ (T−1,T−2,T−3,T−4) electrodes for the first, second, fifth and hundredth cycles with a current density of 100 mAg^−1^. At the first cycle, all the discharge curves of rGO/Fe_2_O_3_ (T−1,T−2,T−3,T−4) gave a first discharge capacity of 1001.1116, 1, 1072.4,9.6 and 1261.5 mAhg^−1^, alternatively. During the second cycle, the discharge capacity decreased to 985.1055, 4, 725.5,.6 and 917.3 mAhg^−1^, alternatively. During the fifth cycle, the discharge capacity decreased to 897.6, 701.3, 984.5 and 868.1 mAhg^−1^, alternatively. The discharge capacities of the rGO/Fe_2_O_3_ (T−1,T−2,T−3,T−4) electrodes amplified up to 327.1, 614.3, 715.3 and 834.1 mAhg^−1^, alternatively, in the 100th cycle.

In order to study the effects of the rGO and MOs’ ratios, ball milling was employed to develop nanocomposites with varying rGO and MOs’ concentrations. Because of the larger amount of rGO than MOs in the T−1,T−2,T−3 and T−4 composites, the MOs’ particles are significantly clustered, which would be deleterious to conductivity. As a result, a large amount of rGO is bare, with only a few MOs attached to it. As a result, the rGO/MOsT−5 composition has the highest cyclability of all the compositions due to the optimal ratio of rGO and MOs (50%:50%). The following are some of the reasons: when the rGO percentage is too low, a large number of MOs form a cluster, resulting in a rapid capacity loss while cycling. However, when the rGO percentage is too high, the capacity is steady but constrained by the rGO’s low theoretical capacity. As a result, the optimal content ratio (rGO and MOs) in the composite rGO/MOsT−5 (50%:50%) strikes a compromise between strong cyclability and high capacity.

In addition, Figure 5d depicts the charge–discharge voltage profiles of the rGO/Co_3_O_4_T−5 electrode for the first, second, fifth and hundredth cycles at a current density of 100 mAg^−1^. The charge/discharge voltage profiles of the rGO/Co_3_O_4_T−5 electrode at the 100 mAg^−1^ current density agreed with the CV results. The sample had a first discharge capacity of 1259.8 mAhg^−1^ but with a significant irreversible loss, such as a Coulombic efficiency of just 63.28%, which is due to the electrolyte solution reactions and the creation of a solid electrolyte interface layer [66]. Despite the significant irreversible loss, the first cycle achieved a substantially higher charge capacity of 797.3 mAhg^−1^. The second cycle achieved an enhanced Coulombic efficiency of 92.62% with a discharge capacity of 861.5 mAhg^−1^ and a charge capacity of 803 mAhg^−1^. The Coulombic efficiency was increased up to 95.81% in the fifth charge–discharge cycle, ensuring improved reversibility. Moreover, it is interesting to note that the irreversible losses after the first charge/discharge cycles were not only due to the trapping of Li-ions inside the metal oxide particles but also the formation of the SEI layer, which played a significant role in the irreversible losses. In addition, the metal oxide/rGO composite materials showed a large, irreversible capacity loss during the initial cycles because of the formation of an SEI, which also plays a key role in the cycle performance and Coulombic efficiency of LIBs [67]. However, all electrode materials for LIBs suffer from degradation phenomena, including solid electrolyte interphase (SEI) layer formation. The SEI layer could serve as a protective layer formed on the anode particle surface, but it becomes unstable during cycling, which accelerates the higher irreversible capacity loss. The SEM images of the rGO/Fe_2_O_3_T−5 and rGO/Co_3_O_4_T−5 electrodes after the initial 25 cycles and 500 cycles are shown in Figure S9 to provide an idea, and the overall electrode structure is stable, but the thin layer of the SEI-layer transformations is hard to observe during these cycling phases. In the meantime, the discharge capacities of the rGO/Fe_2_O_3_T−5 and rGO/Co_3_O_4_T−5 nanocomposites after the 100th cycle (Figure 5c,d) are still much greater than the theoretical capacity of commonly used graphite anodes, e.g., 372 mA h g^−1^. Figure S8 shows the charge–discharge voltage profiles of the rGO/Co_3_O_4_ (T−1,T−2,T−3,T−4) electrodes for the first, second, fifth and hundredth cycles with a current density of 100 mAg^−1^. In the first cycle, all the discharge curves of the rGO/Co_3_O_4_ (T−1,T−2,T−3,T−4) electrodes gave a first discharge capacity of 1108.1108, 1, 1098.5,.3 and 1142.3 mAhg^−1^, alternatively. During the second cycle, the discharge capacity decreased to 884.3, 983.5, 913.3 and 987.2 mAhg^−1^, alternatively. During the fifth cycle, the discharge capacity decreased to 601.1, 902.2, 882.2 and 858.5 mAhg^−1^, alternatively. The discharge capacities of the rGO/Co_3_O_4_ (T−1,T−2,T−3,T−4) electrodes reached up to 212.8, 533.1, 621.1 and 722.1 mAhg^−1^, alternatively, in the 100th cycle.

#### 3.2.3. Cyclic Performance as Anode for LIBs 

**Figure 6:**
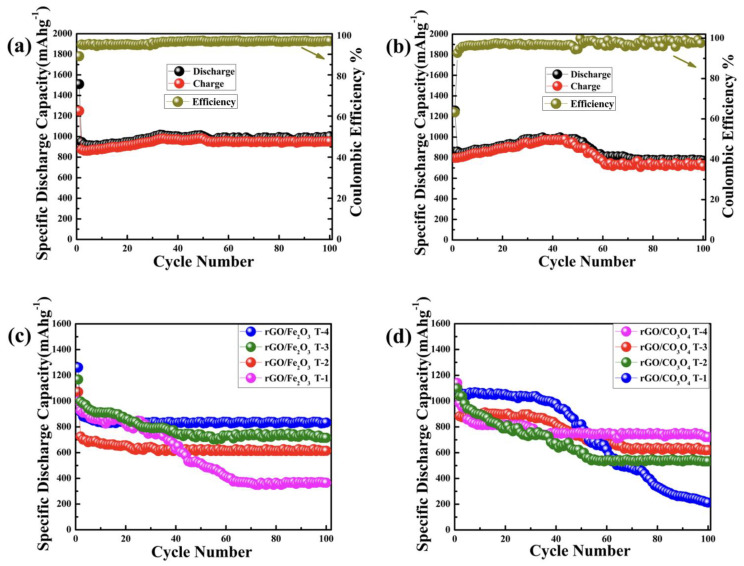
Electrochemical performances of the rGO/Fe_2_O_3_T−5 and the rGO/Co_3_O_4_T−5 anodes: (**a**) cycling performance of the rGO/Fe_2_O_3_T−5 electrode during 100 cycles at a current density of 100 mAg^−1^; (**b**) cycling performance of the rGO/Co_3_O_4_T−5 electrode during 100 cycles at a current density of 100 mAg^−1^. Electrochemical performances of the rGO/Fe_2_O_3_ and the rGO/Co_3_O_4_ anodes with different ratios: (**c**) cycling performances of the rGO/Fe_2_O_3_ electrode with different ratios of rGO and Fe_2_O_3_ during 100 cycles at a current density of 100 mAg^−1^; (**d**) cycling performances of the rGO/Co_3_O_4_ electrodes with different ratios of rGO and Co_3_O_4_ during 100 cycles at a current density of 100 mAg^−1^.

The rGO/Fe_2_O_3_T−5 nanocomposite electrode showcases an outstanding reversible capacity after 100 cycles at a current density of 100 mAg^−1^, as shown in Figure 6a. During the insertion phase, a first discharge capacity of 1510 mAhg^−1^ and a subsequent charge capacity of 1250 mAhg^−1^ were obtained. During the second cycle, the discharge capacity decreased to 861.5 mAhg^−1^ with a corresponding charge capacity of 766 mAhg^−1^, resulting in a substantially greater Coulombic efficiency of 85.3%. Furthermore, in the 100th cycle, the discharge and charge capacities of the rGO/Fe_2_O_3_T−5 nanocomposite rose to 1003.1 and 916 mAhg^−1^, respectively, indicating a probable activation mechanism in the electrode material [65]. The second cycle produced a high discharge capacity of 948.5 mAhg^−1^, which is 94.14% of the theoretical value of Fe_2_O_3_ (1007 mAhg^−1^). The tiny Fe_2_O_3_ particles identified in the TEM studies permitted more effective Li-ion transport, which contributed towards the high specific capacities [68]. Although the capacity declined somewhat over the first ten cycles up to 910 mAhg^−1^, it began to rise after the 11th cycle, which is attributed mostly to the activation process [65]. The specific capacity of the rGO/Fe_2_O_3_T−5 anodes reached 1021.9 mAhg^−1^ in the 100th cycle, enlightening their outstanding cyclability. 

Similarly, Figure 6b indicates the reversible capacity of the rGO/Co_3_O_4_T−5 nanocomposite. The rGO/Co_3_O_4_T−5 nanocomposite delivered a first discharge capacity of 1259.8 mAhg^−1^ but with a large, irreversible loss, e.g., a Coulombic efficiency of only 63.28%, which is related to the reactions of the electrolyte solution and the development of a solid electrolyte interface layer [66]. Despite the large, irreversible loss, a very reasonable charge capacity of 797.3 mAhg^−1^ was still achieved during the first cycle. The increased Coulombic efficiency of 92.62% was achieved in the second cycle, with a discharge capacity of 861.5 mAhg^−1^ and a charge capacity of 803 mAhg^−1^. The Coulombic efficiency was further increased to 95.81% in the third charge–discharge cycle, enlightening better reversibility. The discharge capacity at 100 mAg^−1^ dropped from 861.5 mAhg^−1^ in the second cycle to 773.1 mAhg^−1^ in the 100th cycle. The rGO/Co_3_O_4_T−5 nanocomposite also showcased a fair Li-ion battery anode performance as compared to the reported values for pure cobalt oxide. For example, it was reported that the cobalt oxide shows a specific capacity of 506 mAhg^−1^ during the second cycle and then gradually decreases to 345 mAhg^−1^ at a low current density of 30 mAg^−1^ [69]. Moreover, the cyclic performances of the rGO/Fe_2_O_3_ composite with different ratios of rGO and Fe_2_O_3_ are shown in Figure 6c; the rGO/Fe_2_O_3_T−1, rGO/Fe_2_O_3_T−2, rGO/Fe_2_O_3_T−3 and rGO/Fe_2_O_3_T−4 electrodes deliver capacities of 1001.1169, 1, 1072.4,.3 and 1261.9 mAhg^−1^ in the initial cycle, and retain 365.8, 614.3, 715.3 and 834.1 mAhg^−1^ after 100 cycles, respectively. On the other hand, the cyclic performances of the rGO/Co_3_O_4_ composite with different ratios of rGO and Co_3_O_4_ are shown in Figure 6d; the rGO/Co_3_O_4_T−1, rGO/Co_3_O_4_T−2, rGO/Co_3_O_4_T−3 and rGO/Co_3_O_4_T−4 electrodes deliver capacities of 1088.1108, 8, 1098.5,.2 and 1142.4 mAhg^−1^ in the initial cycle, and retain 212.8, 533.1, 621.1 and 722.1 mAhg^−1^ after 100 cycles, respectively, too. We used a similar procedure to scrutinize the rGO anode. The pristine rGO presented comparatively lower capacities, e.g., 859 mAhg^−1^ during the second cycle and 415.1 mAhg^−1^ after 100 cycles, as shown in Figure S6a, at a current density of 100 mAg^−1^. 

The cycling responses of the rGO/Fe_2_O_3_T−5, rGO/Co_3_O_4_T−5 and rGO anodes at different current rates were evaluated, respectively, as shown in Figure S6b. The rGO/Fe_2_O_3_T−5 anode enlightened a capacity as high as 1509.1 mAhg^−1^ with a current density of 100 mAg^−1^, which changed to 1008 and 937 mAhg^−1^ at a current density of 200 and 500 mAg^−1^, respectively; however, it was maintained up to 1077.2 mAhg^−1^ at a current density of 100 mAg^−1^ after the 20th cycle. Here, the rGO/Fe_2_O_3_T−5 nanocomposite showcased much better performances in terms of the Li-ion storage capacity and cycling stability, as compared to the reported value for the pure Fe_2_O_3_ nanoparticles [70,71,72]. For example, Fe_2_O_3_ nanospheres can provide a fair discharge capacity as high as 800 mAhg^−1^ in the second cycle, but it quickly fades to 414 mAhg^−1^ after 60 cycles at 100 mAhg^−1^, as recently reported [73]. The rGO/Fe_2_O_3_T−5 anode showed an excellent rate capability, as mentioned above. Meanwhile, the rGO/Co_3_O_4_T−5 anode showed a capacity as high as 1276.6 mAhg^−1^ with a current density of 100 mAg^−1^, which changed to 889.9 and 763.2 mAhg^−1^ at a current density of 200 and 500 mAg^−1^, respectively; however, it was maintained up to 949.6 mAhg^−1^ at 100 mAg^−1^ after the 20th cycle. Furthermore, the pristine rGO anode showed a capacity as high as 1113.5 mAhg^−1^ with a current density of 100 mAg^−1^, which changed to 432.9 and 294.5 mAhg^−1^ at a current density of 200 and 500 mAg^−1^, respectively; however, it was maintained up to 473.6 mAhg^−1^ at a current density of 100 mAg^−1^ after the 20th cycle. Moreover, the comparison between the electrochemical performances of various Fe_2_O_3_ and Co_3_O_4_-based composite anodes and the various recently reported electrodes for Li-ion batteries is also shown in Table S1. In addition, metal oxides play a significant role in the higher electrochemical performances of the electrodes. The decorated metal oxides provide massive impacts on the charge–discharge profiles of the electrodes because of the incomplete shell of metal oxides, which provides a wide band gap, enhanced chemical reactivity, electrical conductivity and stability [74]. The decorated metal oxides (MOs) show promising charge and discharge capacities as electrode materials for LIBs because of their rich redox reactions, which involve different ions, contributing to high specific capacities. The nanocomposites with decorated metal oxides not only inherit the advantages of a large electrode/electrolyte contact area, rich charge storage reaction sites, short Li^+^ diffusion path and strong strain adaptability of metal oxides, but also significantly improve the conductivity of the electrode, which is helpful to alleviate the volume change and maintain the stability of the structure. Moreover, the size of the decorated MOs’ particles is very important in enhancing the charge–discharge profiles. The ultra-small decorated MOs nanoparticles and rGO nanosheets give the nanocomposite a reasonably enlarged electroactive surface area due to the synergistic effect of the ultra-high surface area of the rGO and ultra-small MOs nanoparticles, thus maintaining a large capacity, good Coulombic efficiency, high rate capability and cycling stability for the MOs-rGO nanocomposites [38,67]. When metal oxides are decorated on rGO sheets, they can effectively reduce the degree of rGO restacking, maintain their high active surface area and increase the lithium storage capacity and cycle performance. By decorating the metal oxides on rGO, metal oxides can modify the pore characteristics, ion intercalation/deintercalation and conductivity, which lead towards significantly higher charge–discharge profiles of the anodes for LIBs [75]. As for the decorated metal oxides concerned, such as cobalt oxide and iron oxide, they are capable of Li^+^ insertion/extraction in excess of six or eight Li^+^ per formula units, resulting in a significantly large reversible capacity [76]. Thus, it might be concluded that the decoration of the metal oxides on rGO sheets seems to be a good combination since metal oxides hinder rGO from restacking and carry the role of a stabilizer, which prevents the accumulation of rGO sheets and helps to improve the charge–discharge profiles of the electrodes. 

Meanwhile, the pulverization process is often caused by the high-volume tilting created during the Li-ion insertion and extraction process, which causes anode disintegration and, as a result, the loss of electrical connections [77]. Meanwhile, the rGO sheets in the binary samples serve as highly conductive scaffolding to retain the electrical contact from the metal oxides to the current collectors and increase the cycle stability due to their very high electrical conductivity and large surface area [78]. The higher reversible capacities and cyclabilities produced by the rGO/Fe_2_O_3_T−5 and rGO/Co_3_O_4_T−5 nanocomposites were remarkable when compared to other weight ratios and pristine rGO, which could be attributed to the fact that these composites may contain complex features and an optimal ratio of rGO and MOs in the composites. In addition, the SEM images of the nanocomposites, rGO/Fe_2_O_3_T−5 and rGO/Co_3_O_4_T−5, at a low and high current density of 100 mAg^−1^ and 500 mAg^−1^, alternatively are shown in Figure S10 to observe the disintegration phenomenon of the electrodes at lower and higher current rates. The absence of severe disintegration/deformation in both the rGO/Fe_2_O_3_T−5 and rGO/Co_3_O_4_T−5 electrodes at a lower current density of 100 mAg^−1^ can be seen in Figure S10a,c. Meanwhile, the severe disintegration/deformation of both the rGO/Fe_2_O_3_T−5 and rGO/Co_3_O_4_T−5 electrodes can be seen clearly at the high current density of 500 mAg^−1^ in Figure S10b,d, proving that the deformation phenomenon and stresses in both the rGO/Fe_2_O_3_T−5 and rGO/Co_3_O_4_T−5 electrodes materials at high current rates during the electrochemical cycling of Li-ion batteries were present. 

#### 3.2.4. Electrochemical Impedance 

**Figure 7:**
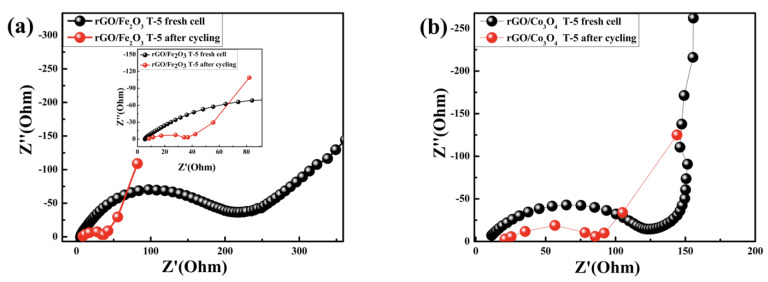
(**a**) EIS results of the rGO/Fe_2_O_3_T−5 electrode before and after 100 cycles; (**b**) EIS results of the rGO/Co_3_O_4_T−5 electrode before and after 100 cycles.

The enhancement of the electronic conductivity of the rGO/Fe_2_O_3_T−5 nanocomposite was confirmed by the electrochemical impedance spectroscopy measurements, which were carried out with working electrodes before and after the cycling. Figure 7a,b shows the Nyquist plots for the half-cells with the rGO/Fe_2_O_3_T−5 and rGO/Co_3_O_4_T−5 electrodes before and after the cycling, respectively, in which both show a semicircle at the high- to middle-frequency region, followed by an inclining line at the low-frequency region. It is well known that the size of the semicircle in the mid-frequency region is a sign of the charge-transfer resistance (Rct) in the electrode reaction, and the inclined line in the low frequency enlightens the Warburg impedance (Zw) related to lithium diffusion in the solid [79]. From the Nyquist plots, it can be observed that the diameter of the semicircle for the rGO/Fe_2_O_3_T−5 electrode in the mid-frequency region is smaller than that of the rGO/Co_3_O_4_T−5, which reveals that the charge-transfer resistances of the rGO/Fe_2_O_3_T−5 electrode are smaller than that of the rGO/Co_3_O_4_T−5 electrode. This was owing to the highest dispersion of iron oxide particles in the rGO matrix, which may have benefitted the rGO/Fe_2_O_3_T−5 electronic conductivity after milling. 

## 4. Conclusions

We developed an ecologically friendly and scalable approach for synthesizing metal oxides, such as the Fe_2_O_3_ and Co_3_O_4_ nanoparticles, wrapped by reduced graphene oxide (rGO) sheets, to construct hybrid nanocomposites. When tested as Li-ion battery anodes, these MO/rGO nanocomposites showcased high specific capacities and excellent cyclabilities in the 100th cycle, e.g., the rGO/Fe_2_O_3_T−5 and rGO/Co_3_O_4_T−5 composites attained 1021 and 773 mAhg^−1^ at 100 mAg^−1^, respectively, which are higher than that of the pristine rGO anode (415 mAhg^−1^ at a current density of 100 mAg^−1^ after 100 cycles). An approach of this adaptability may be used for other transition metal oxides, opening up new paths for the development of new electrode materials for rechargeable LIBs.

## Figures and Tables

**Table 1:** Composition of each anode material.

Weight Ratio %					
Sample Code	rGO	Fe_2_O_3_	Sample Code	rGO	Co_3_O_4_
rGO/Fe_2_O_3_T−1	90	10	rGO/Co_3_O_4_T−1	90	10
rGO/Fe_2_O_3_T−2	80	20	rGO/Co_3_O_4_T−2	80	20
rGO/Fe_2_O_3_T−3	70	30	rGO/Co_3_O_4_T−3	70	30
rGO/Fe_2_O_3_T−4	60	40	rGO/Co_3_O_4_T−4	60	40
rGO/Fe_2_O_3_T−5	50	50	rGO/Co_3_O_4_T−5	50	50

## Data Availability

The data presented in this study are available on request from the corresponding authors.

## Supplementary Materials

The following supporting information can be downloaded at: https://www.mdpi.com/article/10.3390/nano13020296/s1. Figure S1: (a) Nitrogen adsorption–desorption isotherms of the rGO/Co_3_O_4_T−5 nanocomposite and (b) the corresponding pore size distribution of the rGO/ Co_3_O_4_T−5 nanocomposite.; Figure S2: The TGA curves of the rGO/Fe_2_O_3_T−5 and rGO/Co_3_O_4_T−5 nanocomposites.; Figure S3: (a,b) SEM images of the nanocomposites rGO/Fe_2_O_3_T−5 and the nanocomposite rGO/Co_3_O_4_T−5, alternatively, after 100 charge–discharge cycles.; Figure S4: (a) SEM image of the nanocomposite rGO/Fe_2_O_3_T−5 and the corresponding elemental mapping of (b) Fe, (c) C, (d) O, (e) SEM image of the nanocomposite rGO/Co_3_O_4_T−5, and the elemental mapping of (f) Co, (g) C, (h) O.; Figure S5: (a) SEM image of rGO, (b) TEM image of rGO and the corresponding elemental mapping of (c) C, (d) O.; Figure S6: (a) Cycling performance and Coulombic efficiency of the pristine rGO electrode at 100 mAg^−1^ current density during 100 cycles, (b) rate performance and the Coulombic efficiencies of the rGO/Fe_2_O_3_T−5, rGO/Co_3_O_4_T−5 and rGO electrodes at various current densities between 100 mAg^−1^ and 500 mAg^−1^.; Figure S7: Galvanostatic charge–discharge profiles: (a–d) the rGO/Fe_2_O_3_ electrodes at 1st, 2nd, 5th and 100th cycles with different ratios of rGO and Fe_2_O_3_ at the current density of 100 mAg^−1^.; Figure S8: Galvanostatic charge–discharge profiles: (a–d) the rGO/Co_3_O_4_ electrodes at 1st, 2nd, 5th and 100th cycles with different ratios of rGO and Co_3_O_4_ at the current density of 100 mAg^−1^.; Figure S9: (a,b) SEM images of the nanocomposite rGO/Fe2O3T−5 at a low current density of 100 mAg^−1^ after 25 cycles and 500 cycles, (c,d) SEM images of the nanocomposite rGO/Co3O4T−5 at a low current density of 100 mAg^−1^ after 25 cycles and 500 cycles, alternatively.; Figure S10: (a,b) SEM images of the nanocomposite rGO/Fe_2_O_3_T−5 at a low current density of 100 mAg^−1^ and at a high current density of 500 mAg^−1^ and (c,d) SEM images of the nanocomposite rGO/Co_3_O_4_T−5 at a low current density of 100 mAg^−1^ and at a high current density of 500 mAg^−1^, alternatively, after 100 charge–discharge cycles.; Table S1: Comparison of the electrochemical performances of Fe_2_O_3_ and Co_3_O_4_-based composite anodes and various recently reported electrodes for Li-ion batteries. References [80,81,82,83,84,85,86,87] are cited in the supplementary materials.
