# Supplementary material for: Metal Oxide Wrapped by Reduced Graphene Oxide Nanocomposites as Anode Materials for Lithium-Ion Batteries
Source: Nanomaterials (Basel). 2023 Jan 11;13(2):296. doi: 10.3390/nano13020296 (PMC9865346; doi:10.3390/nano13020296)
Supplement: Supplementary file 1 [file nanomaterials-13-00296-s001.zip › nanomaterials-2148321-supplementary.pdf]

Supplementary Information

Metal oxide wrapped by reduced graphene oxide nanocomposites as anode materials for lithium-ion batteries

Junaid Aslam[1], Yong Wang[1,2]*

[1] Department of Chemical Engineering, School of Environmental and Chemical Engineering, Shanghai University, 99 Shangda Road, Shanghai 200444, People's Republic of China.

[2] Key Laboratory of Organic Compound Pollution Control Engineering (MOE), Shanghai University, 99 Shangda Road, Shanghai 200444, People's Republic of China

*Corresponding authors: Tel: +86-21-66137723; fax: +86-21-66137725.

Email address: Yong Wang (yongwang@shu.edu.cn)

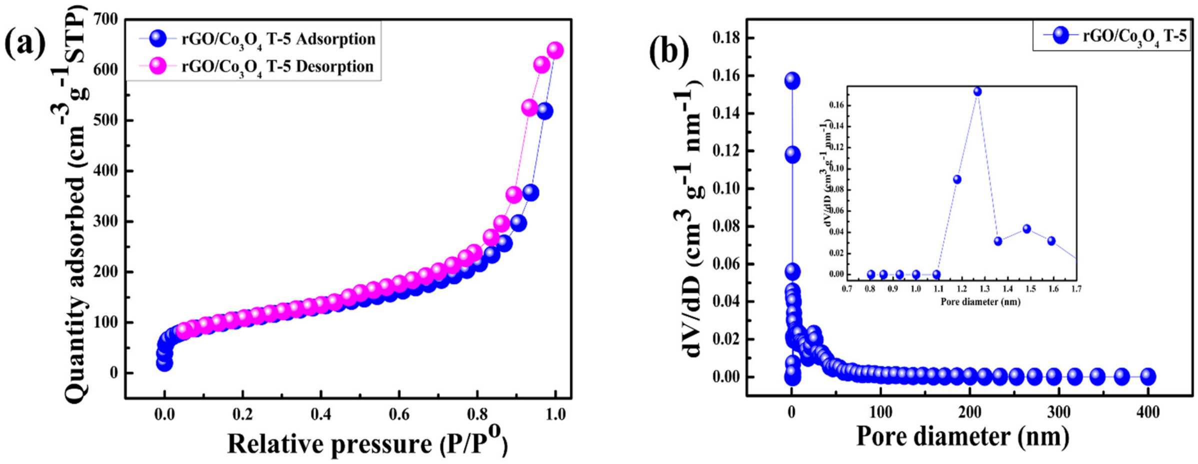

Figure S1. (a) Nitrogen adsorption-desorption isotherms of the rGO/Co_3O_4T–5 nanocomposite and (b) the corresponding pore-size distribution of the rGO/Co_3O_4T–5 nanocomposite.

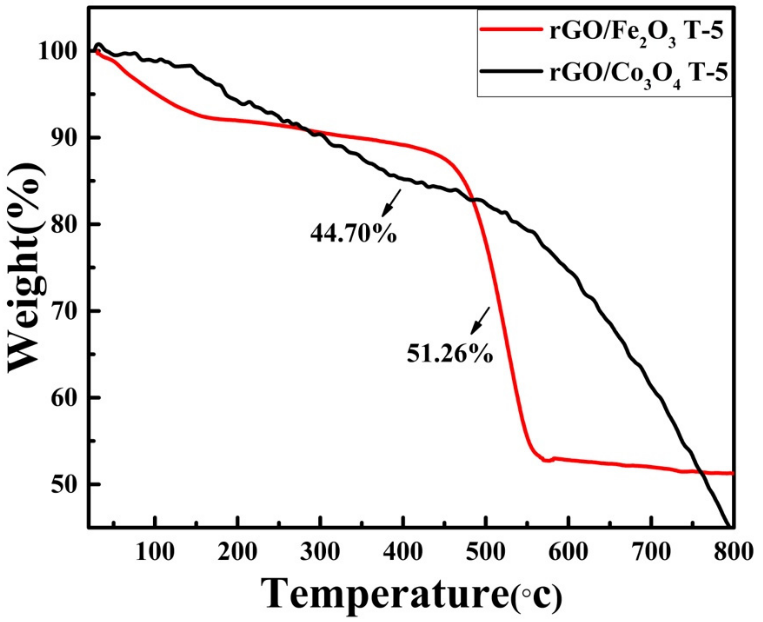

Figure S2. The TGA curves of the rGO/Fe_2O_3T−5 and rGO/Co_3O_4T−5 nanocomposites.

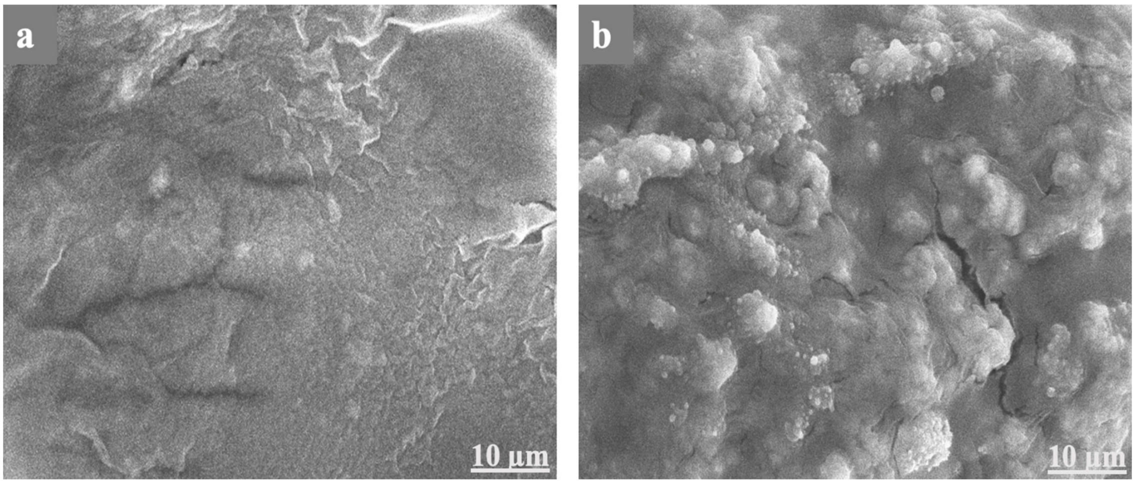

Figure S3. (a, b) SEM images of the nanocomposites rGO/Fe_2O_3T−5 and the nanocomposite rGO/Co_3O_4T−5 alternatively, after 100 charge–discharge cycles.

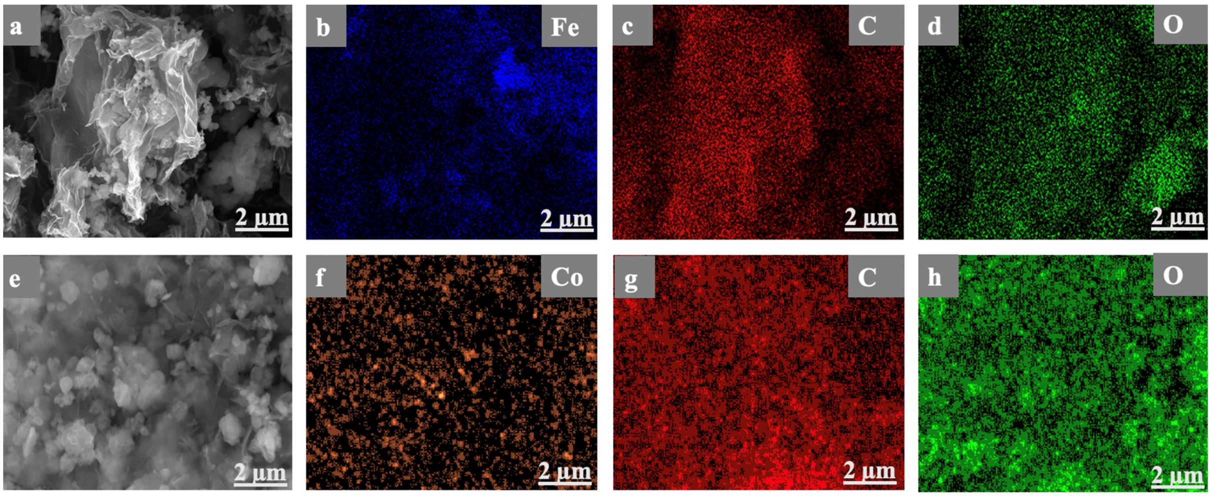

Figure S4. (a) SEM image of the nanocomposite rGO/Fe_2O_3T−5, and the corresponding elemental mapping of (b) Fe , (c) C, (d) O, (e) SEM image of the nanocomposite rGO/Co_3O_4T−5, and the elemental mapping of (f) Co , (g) C, (h) O.

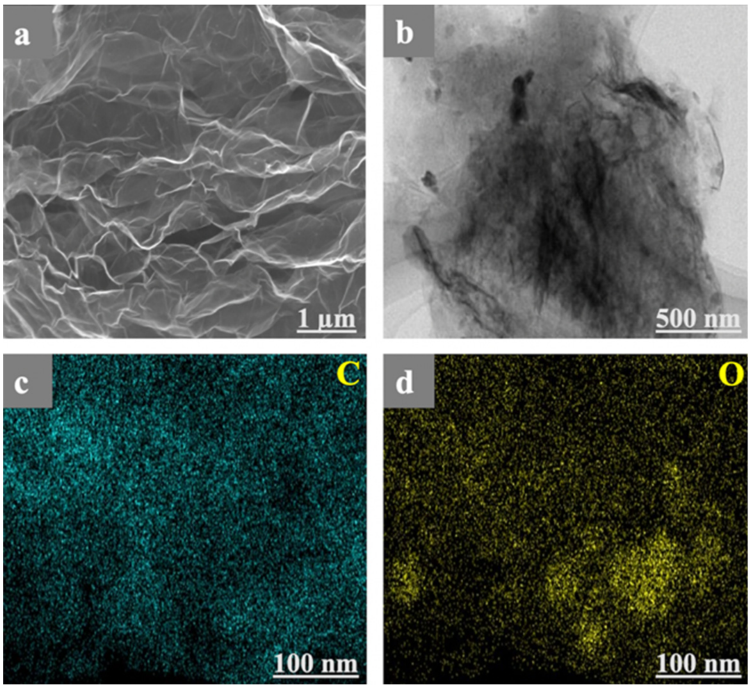

Figure S5. (a) SEM image of rGO, (b) TEM image of rGO and the corresponding elemental mapping of (c) C, (d) O.

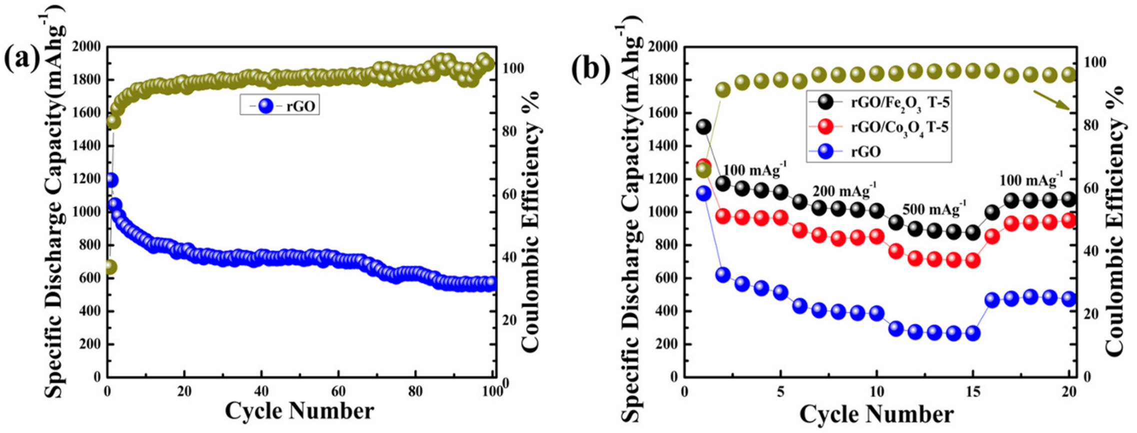

Figure S6. (a) Cycling performance and coulombic efficiency of the pristine rGO electrode at 100 mAg^{-1} current density during 100 cycles (b) Rate performance and the coulombic efficiencies of the rGO/Fe_2O_3T–5, rGO/Co_3O_4T–5 and rGO electrodes at various current densities between 100 and 500 mAg^{-1}.

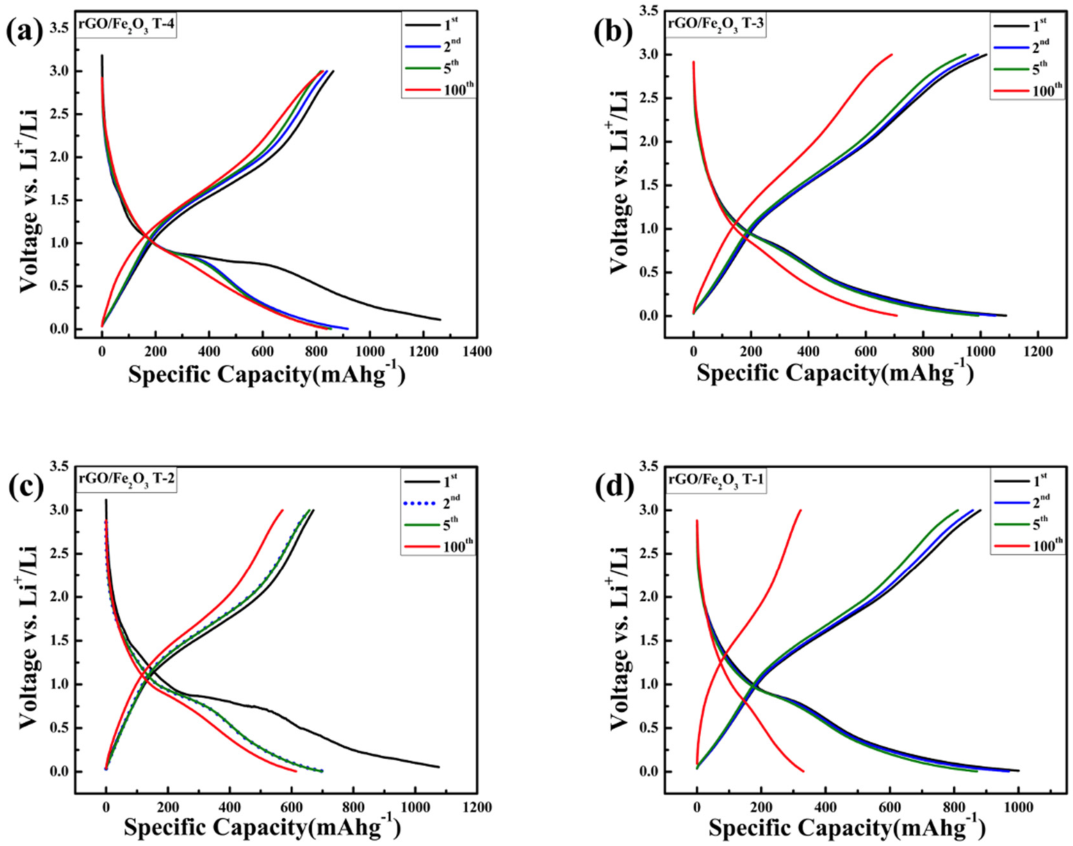

Figure S7. Galvanostatic charge-discharge profiles: (a-d) The rGO/Fe_2O_3 electrodes at 1st, 2nd, 5th and 100th cycles with different ratios of rGO and Fe_2O_3 at the current density of 100 mAg^{-1}.

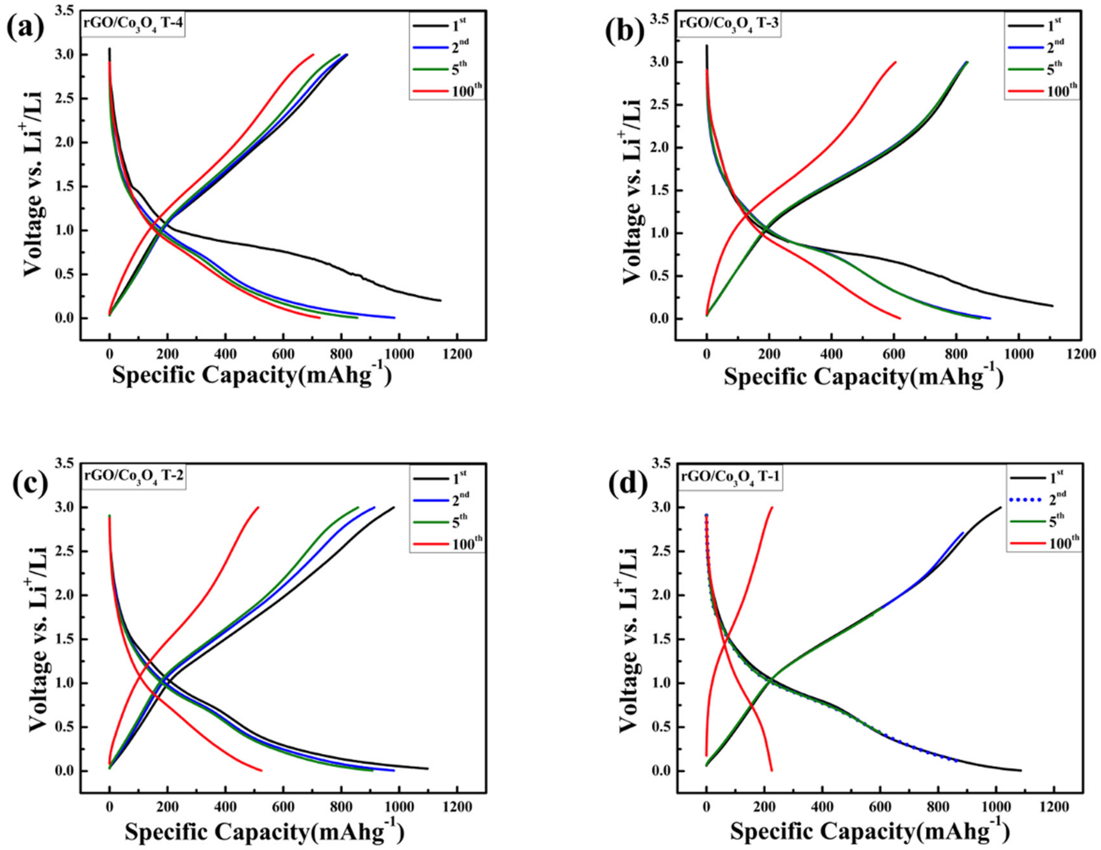

Figure S8. Galvanostatic charge-discharge profiles: (a-d) The rGO/Co_3O_4 electrodes at 1st, 2nd, 5th and 100th cycles with different ratios of rGO and Co_3O_4 at the current density of 100 mAg^{-1}.

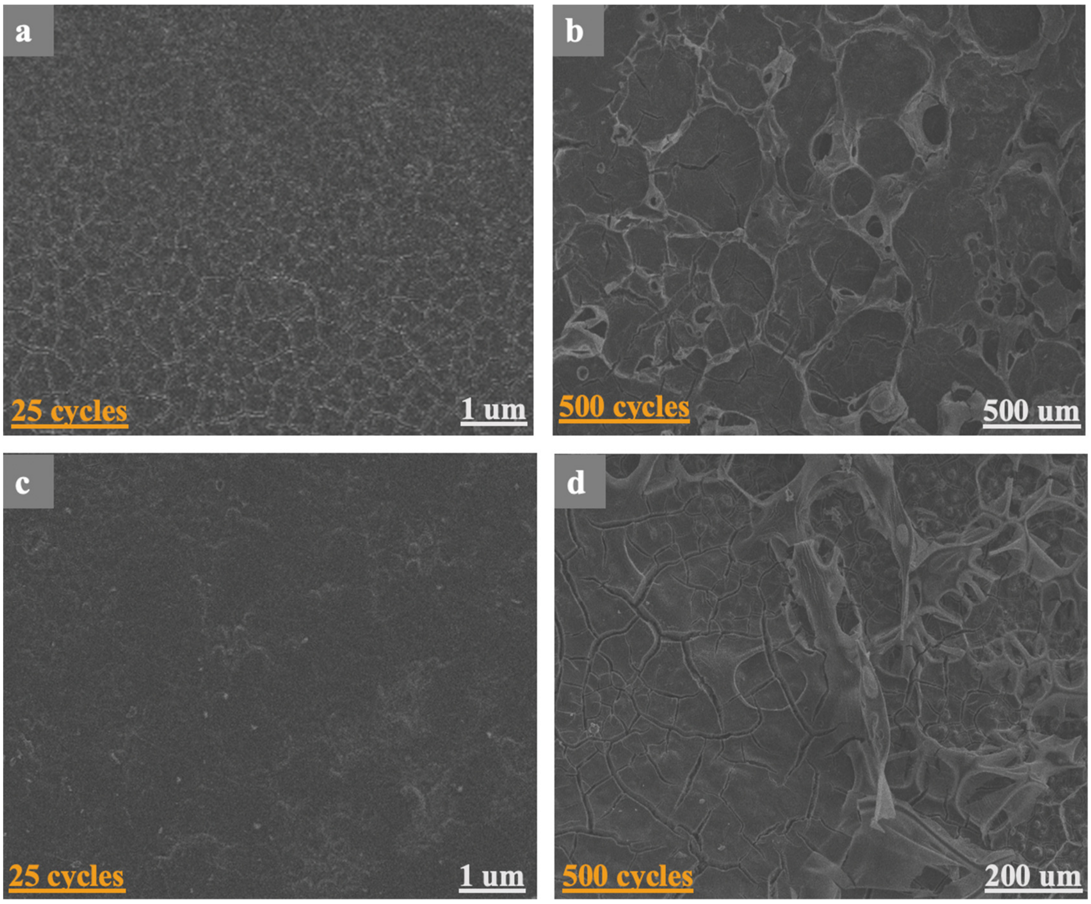

Figure S9. (a, b) SEM images of the nanocomposite rGO/Fe_2O_3T−5 at a low current density 100 mAg^{-1} after 25 cycles and 500 cycles (c, d) SEM images of the nanocomposite rGO/Co_3O_4T−5 at a low current density 100 mAg^{-1} after 25 cycles and 500 cycles alternatively.

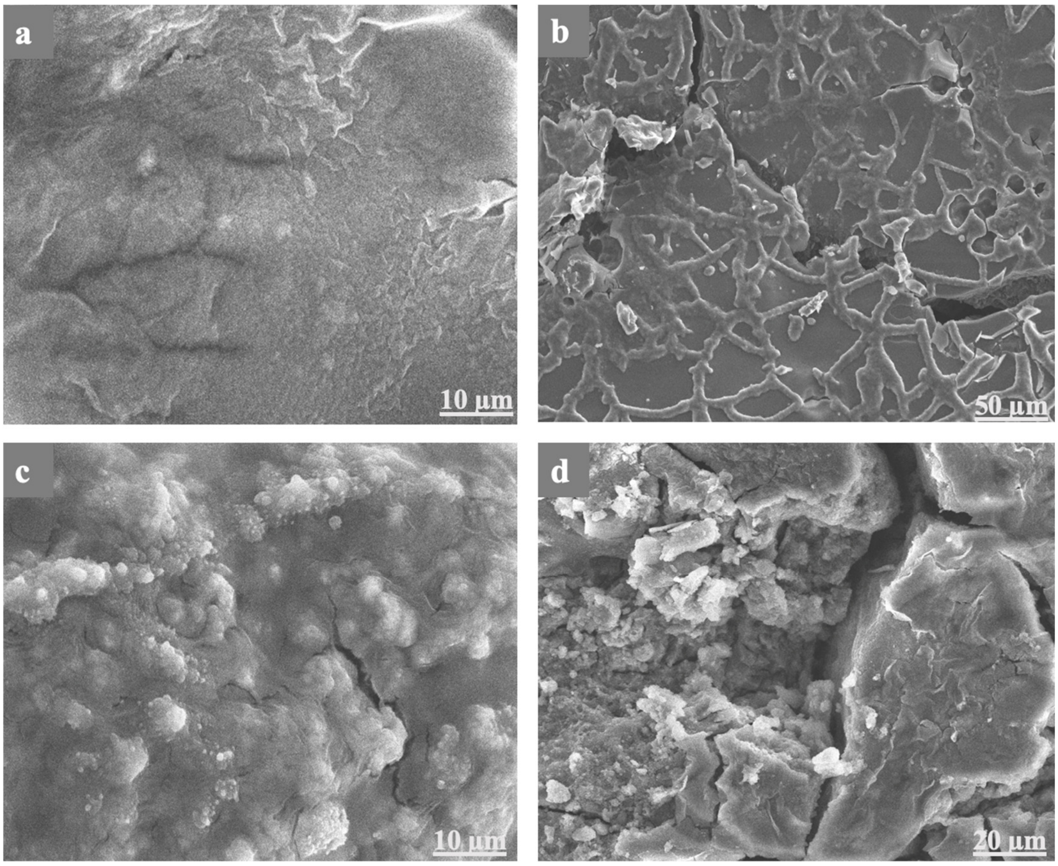

Figure S10. (a, b) SEM images of the nanocomposite rGO/Fe_2O_3T−5 at low current density 100 mAg^{-1} and at high current density 500 mAg^{-1} and (c, d) SEM images of the nanocomposite rGO/Co_3O_4T−5 at low current density 100 mAg^{-1} and at high current density 500 mAg^{-1} alternatively, after 100 charge–discharge cycles.

Table S1. Comparison of the electrochemical performances of Fe_2O_3 and Co_3O_4 based composite anodes and various recently reported electrodes for Li-ion batteries.

Electrode materials	Current Density (mAg^{-1})	Cycle Number (nth)	Reversible Capacity ($mAhg^{-1}$)	Ref.
α-Fe_2O_3 nanofiber	60	40	1293	[1]
α- Fe_2O_3 nanocapsules	0.1	30	740	[2]
Fe_2O_3@C composites	200	100	600	[3]
RG-O/Fe_2O_3 composite	100	50	1027	[4]
Co_3O_4/RGO	100	100	96.36	[5]
Co_3O_4-graphene sheet-on-sheet nanocomposite	89	30	1065	[6]
Co_3O_4/graphene	0.05	30	935	[7]
Mesoporous Co_3O_4/Graphene	100	35	820	[8]
Co_3O_4/graphene	100	50	840	[9]
Co_3O_4 fibers/graphene	100	40	840	[10]
rGO/Fe_2O_3T−5<br>rGO/ Co_3O_4T−5	100<br>100	100<br>100	1021<br>773	This Work<br>This Work

References:

1. S. Chaudhari and M. Srinivasan, "1D hollow α-Fe_2O_3 electrospun nanofibers as high performance anode material for lithium ion batteries," *Journal of Materials Chemistry*, vol. 22, no. 43, pp. 23049-23056, 2012.
2. H. S. Kim, Y. Piao, S. H. Kang, T. Hyeon and Y.-E. Sung, "Uniform hematite nanocapsules based on an anode material for lithium ion batteries," *Electrochemistry communications*, vol. 12, no. 3, pp. 382-385, 2010.
3. F. Han, D. Li, W. C. Li, C. Lei, Q. Sun and A. H. Lu, "Nanoengineered polypyrrole-coated Fe_2O_3@C multifunctional composites with an improved cycle stability as lithium-ion anodes," *Advanced Functional Materials*, vol. 23, no. 13, pp. 1692-1700, 2013.
4. X. Zhu, Y. Zhu, S. Murali, M. D. Stoller and R. S. Ruoff, "Nanostructured reduced graphene oxide/Fe_2O_3 composite as a high-performance anode material for lithium ion batteries," *ACS nano*, vol. 5, no. 4, pp. 3333-3338, 2011.
5. D. Pan, S. Wang, B. Zhao, M. Wu, H. Zhang, Y. Wang and Z. Jiao, "Li storage properties of disordered graphene nanosheets," *Chemistry of Materials*, vol. 21, no. 14, pp. 3136-3142, 2009.
6. S. Q. Chen and Y. Wang, "Microwave-assisted synthesis of a Co_3O_4–graphene sheet-on-sheet nanocomposite as a superior anode material for Li-ion batteries," *Journal of Materials Chemistry*, vol. 20, no. 43, pp. 9735-9739, 2010.
7. Z.-S. Wu, G. Zhou, L.-C. Yin, W. Ren, F. Li and H.-M. Cheng, "Graphene/metal oxide composite electrode materials for energy storage," *Nano Energy*, vol. 1, no. 1, pp. 107-131, 2012.
8. X. Yang, K. Fan, Y. Zhu, J. Shen, X. Jiang, P. Zhao and C. Li, "Tailored graphene-encapsulated mesoporous Co_3O_4 composite microspheres for high-performance lithium ion batteries," *Journal of Materials Chemistry*, vol. 22, no. 33, pp. 17278-17283, 2012.
9. X. Chi, L. Chang, D. Xie, J. Zhang and G. Du, "Hydrothermal preparation of Co_3O_4/graphene composite as anode material for lithium-ion batteries," *Materials Letters*, vol. 106, pp. 178-181, 2013.
10. X. Yang, K. Fan, Y. Zhu, J. Shen, X. Jiang, P. Zhao, S. Luan and C. Li, "Electric papers of graphene-coated Co_3O_4 fibers for high-performance lithium-ion batteries," *ACS applied materials & interfaces*, vol. 5, no. 3, pp. 997-1002, 2013.
